# Isolated splenic tuberculosis with subsequent paradoxical deterioration: a case report

**DOI:** 10.1186/s13104-017-2483-2

**Published:** 2017-04-24

**Authors:** Frederick Wangai, Loice Achieng, George Otieno, Jacqueline Njoroge, Tabitha Wambaire, Jamilla Rajab

**Affiliations:** 1 Department of Clinical Medicine and Therapeutics, School of Medicine, College of Health Sciences-University of Nairobi, P.O. Box 19676, Nairobi, 00202 Kenya; 2 Haematology and Blood Transfusion Unit, Department of Human Pathology, School of Medicine, College of Health Sciences-University of Nairobi, P.O. Box 19676, Nairobi, 00202 Kenya

**Keywords:** Isolated tuberculosis of spleen, Splenic tuberculosis, Tuberculosis, Spleen, Paradoxical reaction, Case report, GeneXpert, Core needle biopsy, Fine needle aspiration cytology

## Abstract

**Background:**

Isolated tuberculosis of the spleen has been described occasionally in literature, mostly in immunosuppressed individuals with various risk factors. Sequestration in the spleen makes such *Mycobacterium tuberculosis* infection difficult to diagnose. This report describes an extremely rare case of isolated splenic tuberculosis in an immunocompetent individual.

**Case presentation:**

A 26 year old Kenyan male presented with pyrexia of unknown origin, with negative screening tests for bacterial, fungal and parasitic infections. Ziehl–Neelsen staining and GeneXpert tests were negative for *M. tuberculosis*. Diagnosis of isolated splenic tuberculosis was made on core biopsy of the spleen. The patient initially worsened upon treatment with antituberculous medication attributable to the ‘Paradoxical Reaction’ phenomenon, before making full recovery.

**Conclusions:**

This case highlights the need to continuously be on the lookout for tuberculosis especially in unusual presentations, including subsequent paradoxical reaction which may be encountered.

**Electronic supplementary material:**

The online version of this article (doi:10.1186/s13104-017-2483-2) contains supplementary material, which is available to authorized users.

## Background

Isolated tuberculosis of the spleen in immunocompetent hosts is rare [1–3]. Splenic involvement is more frequently reported in cases of disseminated tuberculosis following haematogenous spread of *Mycobacterium tuberculosis* (MTB) and in patients with significant immune suppression [4, 5]. However, there are few reports of isolated splenic tuberculosis worldwide [1, 5–9]. Risk factors associated with tuberculosis (TB) of the spleen include immunosuppression, preceding pyogenic infections, splenic abnormalities, prior trauma to the spleen, sickle cell disease and other haemopathies, and in the immune competent patient where another body site has been infected by MTB [6]. An immune competent individual with no prevailing risk factors may prove to be a diagnostic conundrum, as identification of microbial aetiology sequestered in isolated sites such as the spleen is difficult and misdiagnosis is common [9]. In this article we present a previously healthy and immunocompetent young man with isolated splenic TB presenting as pyrexia of unknown origin, and subsequent paradoxical worsening of symptoms upon initiation of antituberculous (anti-TB) therapy.

## Case presentation

A 26 year old Kenyan male presented with a 1-month history of fever. The fever was intermittent, often at night ranging from 38.3 to 40.2 °C, with accompanying prodromal chills and sweats. There was reported unintentional weight loss of 3 kg over a 3 month period. He reported a transient productive cough at the beginning of his illness that subsequently resolved. His medical history did not reveal any allergies, occupational exposure to airway irritants, and did not include asthma, TB or human immunodeficiency virus (HIV) infection. He denied any history of recent significant travel to areas of endemic malaria or arboviral infections. He did not report any bites or stings, had no history of rash and had no pets. He had no sick contacts nor had he handled any animal remains or waste. He had no musculoskeletal complaints, urinary or gastrointestinal symptoms.

A month prior to his presentation at our tertiary referral hospital, he had been admitted to a smaller outside facility with the same symptoms. Investigations done in that facility included a chest radiograph that was normal, sputum smear that was negative for acid-fast-bacilli (AFB) and a negative HIV 4th generation enzyme linked immunosorbent assay (ELISA). He presented to our facility due to worsening of his condition, with increasing frequency of his febrile episodes.

On admission, his physical examination revealed a young man in fair general condition, and of good nutritional status. He was febrile at 40 °C, had tachycardia and normal blood pressure. He had no pallor, icterus, lymph node enlargement or rash. There was no tenderness over his sinuses, and the rest of his head, ear, eye, nose and throat exam was normal. He was not in respiratory distress, his chest was found to be symmetric with normal vesicular breath sounds. Cardiovascular examination did not reveal any murmurs and he had no tenderness nor organomegaly on abdominal examination. His neck was supple and he had no focal neurologic signs. There were no tender or swollen joints.

The complete blood count (CBC), showed leukopenia of 2.53 × 10^9^ cells/L (neutrophils 1.87 × 10^9^ cells/L, lymphocytes 0.536 × 10^9^ cells/L) with a microcytic hypochromic anaemia of 9.43 g/dL (Mean Corpuscular Volume 74.7 fL) and a thrombocytosis of 717 × 10^9^ platelets/L. The C-reactive protein was elevated at 137.7 mg/L (reference <5.0), erythrocyte sedimentation rate 52 mm/h (reference 1–15) and serum ferritin 911.6 ng/mL (reference 34–310). Renal and liver function tests were normal. Successive blood, urine and stool cultures yielded no bacterial growth. MTB was not detected in sputum Ziehl–Neelsen (ZN) smears and GeneXpert MTB/RIF tests performed in our facility. A repeat chest radiograph was normal. At this point, the patient was put on 1 g Paracetamol thrice daily for its antipyretic effect, as further tests investigating possible foci of infection were underway. However, the fevers continued to persist.

**Fig. 1:**
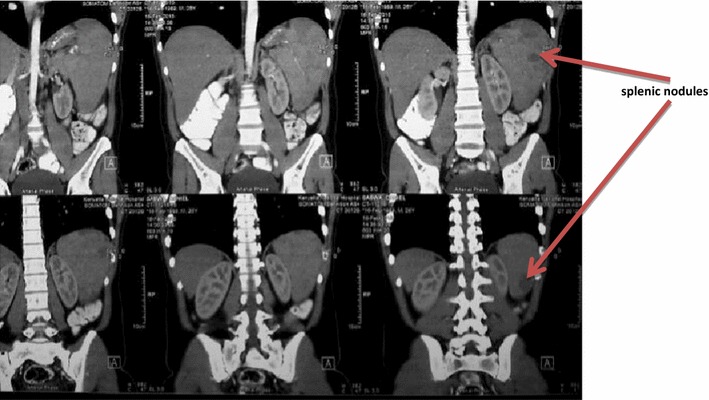
Computed tomography of the abdomen slices showing multiple splenic nodules

Serological tests for HIV, hepatitis B and C, *Salmonella*, *Brucella*, Malaria blood smears and antigen tests were all negative. Fungal blood cultures were negative. He had a normal transthoracic echocardiogram. Connective tissue screening including antinuclear antibodies, rheumatoid factor, extractable nuclear antigens (dsDNA, Sm, Rib-P, U1RNP, Ro, La, CENP, Scl-70, PM-Scl, Fibrillarin, RNA Pol III, Jo-1, Mi-2, PCNA) were all negative. Bone marrow aspirate and trephine biopsy revealed low normocellular marrow with trilineage dysplasia and megakaryocytic hyperplasia. There were no organisms, tumour cells or granulomata demonstrated in the aspirate. Apart from the persistent fevers, the patient while in the ward developed abdominal pain of gradual onset, localised to left upper quadrant. Abdominal ultrasound of the patient revealed an enlarged liver with normal texture. The biliary and hepatic radicles were normal. Notably, the spleen was enlarged (15.66 cm) with a 2.04 × 1.99 cm hypoechoic mass. Computed tomography (CT) scan of the abdomen confirmed a moderately enlarged liver with homogenous echopattern, an enlarged spleen with multiple hypodense non-enhancing nodules, the largest being 2.4 cm in diameter (Fig. 1). A tentative diagnosis of multiple splenic microabscesses was made. Ultrasound-guided fine needle aspiration cytology (FNAC) of one of the splenic lesions was bloody in appearance, revealing scanty leucocytes, some erythrocytes and did not demonstrate any AFB or fungal elements. No bacterial or fungal growths were cultured from the aspirate.

**Fig. 2:**
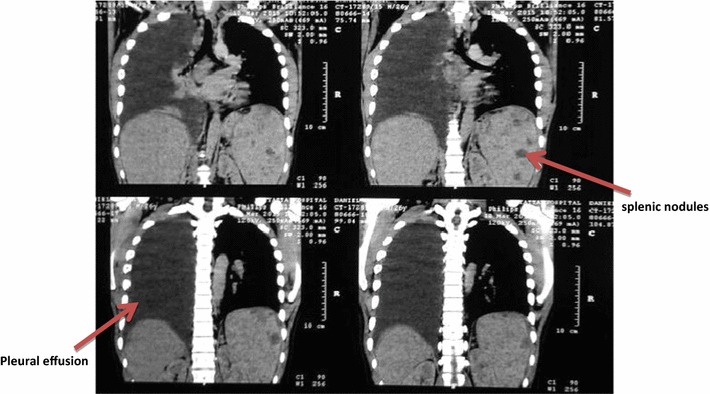
Chest computed tomography slices showing massive right pleural effusion and multiple splenic nodules

During the second month of admission, the patient’s fevers became more frequent, and he experienced gradual increase in abdominal pain. There was marked tenderness in the left upper quadrant with a palpable spleen. In view of worsening neutropenia with increasing abdominal pain and fevers in the background of the abdominal CT scan report citing possible microabscesses, a clinical decision by the Infectious Disease specialists was made to empirically cover for bacterial, fungal and MTB infection as a conclusive diagnosis was yet to be clinched. A broad-spectrum antibiotic cover of intravenous vancomycin 1 g 8 h, ceftazidime 1 g 8 h and metronidazole 500 mg 8 h was prescribed. This was done to provide adequate bacterial gram positive and negative cover as well as to cater for possible anaerobes respectively, as informed by the Clinical Practice Guidelines for Febrile Neutropaenia [10, 11]. Empiric antifungal of choice was amphotericin-B and the patient was initiated on anti-TB therapy with rifampin, isoniazid, ethambutol and pyrazinamide. About a week after initiation of the antimicrobial therapy, the patient deteriorated further with a worsening leukopenia of 1.06 × 10^9^ cells/L with a neutrophil count of 0.004 × 10^9^ cells/L, lymphocyte count of 0.505 × 10^9^ cells/L, monocyte count of 0.511 × 10^9^ cells/L, erythrocyte count of 3.79 × 10^12^ cells/L and platelet count of 443 × 10^9^/L. He had worsening of fevers, cough of new onset, difficulty in breathing, chest and abdominal pain. Examination revealed a right pleural effusion which was confirmed on chest radiograph. Chest CT revealed massive right pleural effusion, right mid and lower zone consolidation and multiple paratracheal and mediastinal lymph nodes (Fig. 2). He had drainage of the effusion with a sample taken for analysis. Results showed an exudate demonstrating a heterogeneous population of mature and immature lymphocytes within a proteinaceous haemorrhagic background, as well as normal adenine deaminase levels. Gram stain for bacteria and ZN stain for AFB were both negative. The pleural fluid had no malignant cells.

**Fig. 3:**
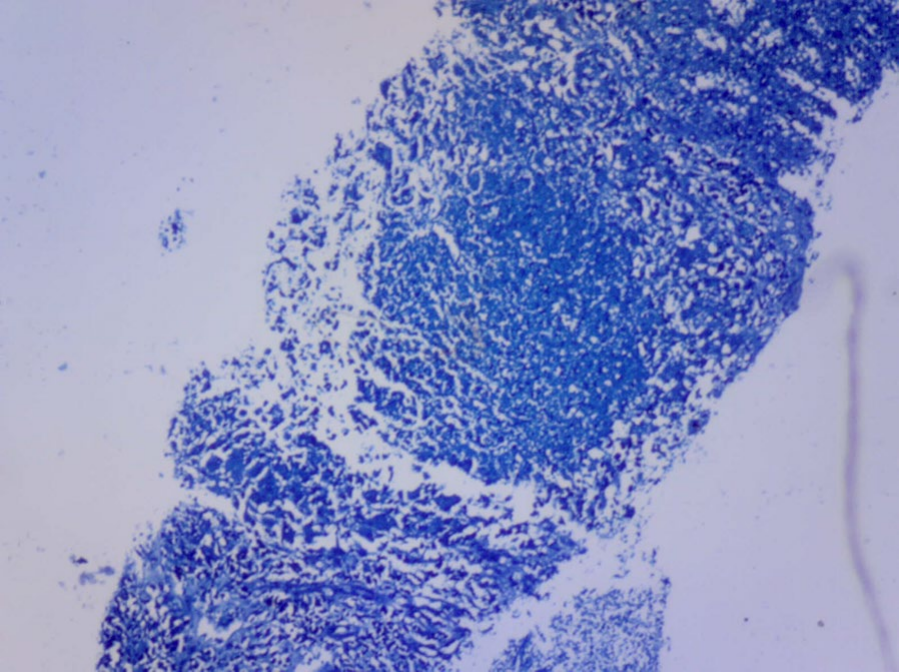
Splenic biopsy showing granulomas

**Fig. 4:**
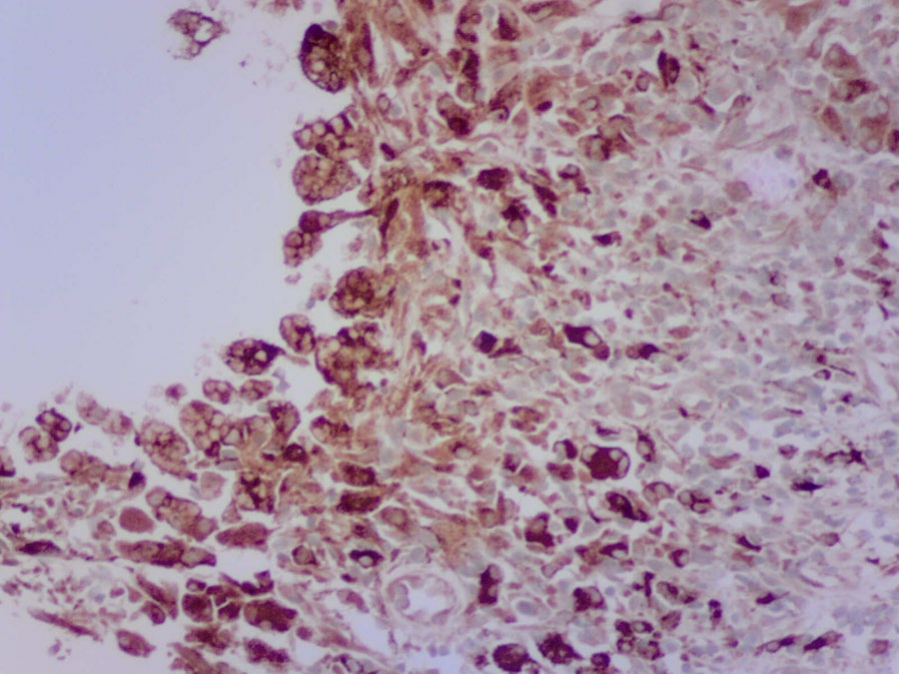
CD68 stain showing positive histiocytes in granuloma

**Fig. 5:**
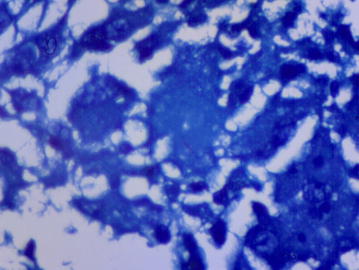
Ziehl–Neelsen stain positive for acid-fast bacilli

A percutaneous image-guided core needle biopsy (CNB) of his spleen was performed. This involved splenic sampling using a cutting needle under guidance of ultrasound. Histopathological examination of the biopsy specimen revealed effaced splenic architecture with extensive multiple granulomas—mainly necrotising with multinucleate histiocytes, eosinophils and areas of fibrosis. There was no evidence of a malignant lymphocytic or foreign cell infiltrate. ZN stain of the biopsy processed specimen revealed acid-fast bacilli (Figs. 3, 4, 5), confirmed by GeneXpert MTB/RIF which yielded MTB sensitive to rifampicin. With this tissue diagnosis confirmed, all the empiric antibiotics (vancomycin, ceftazidime, metronidazole) and antifungals (amphotericin-B) were discontinued and the anti-TB treatment maintained with supportive management comprising analgesics, fluids and nutritional support. In view of the patient’s febrile neutropaenia, the clinicians instituted strict infection control measures such as barrier nursing, hand washing and avoiding raw uncooked foods. These measures were taken in order to protect the patient from acquiring opportunistic infections from his environment, as he was severely neutropaenic thus immunologically vulnerable. The patient continued to deteriorate with persistent neutropaenia and fever for about 2 weeks before he registered progressive improvement and was discharged 30 days after initiation of anti-TB medication. His hospital stay lasted a total of 78 days. It was noted during the patient’s regular follow-up sessions as an outpatient that he remained neutropaenic for about a month’s duration after discharge from the hospital. This neutropaenia resolved about 2 months into the TB treatment with a normal CBC. No adverse events were reported. He subsequently did well and is back to work, having completed a full 6-month course of anti-TB medication.

## Discussion

According to the World Health Organization (WHO) Global Tuberculosis Report released in 2016, TB remains one of the top 10 leading causes of morbidity and death worldwide, even surpassing mortality attributable to HIV annually [12]. This report states that in the year 2015, 10.4 million people were infected with TB globally, and 1.4 million deaths were attributed to this disease. WHO surveillance data shows that TB is a major problem in the African region. Although the continent claimed 26% of the world’s new cases in the same year, it alarmingly had the most severe burden relative to population, that is 275 cases for every 100,000 persons. This figure surpassed more than double the global average of 142 per 100,000 [12]. Our country Kenya is currently classified as one of the 30 high TB burden countries, which collectively account for 87% of all estimated incident cases worldwide. In the year 2015, Kenya’s estimated incidence rate of TB was 233 per 100,000 population, [12] and this disease has been noted to rank as one of the leading causes of mortality in the country [13, 14]. In view of the above, one recognises the importance and relevance of TB to clinicians and public health services in our local setting, regionally and globally at large.

This case report highlights a rare instance of primary splenic tuberculosis. The spleen is one of the organs involved in extrapulmonary TB. However, more common extrapulmonary sites include the lymph nodes, pleura, genitourinary tract, bones and joints, meninges, peritoneum and pericardium [15].

Haematogenous dissemination occurs frequently in HIV-infected individuals. Isolated splenic involvement is rare, and is usually reported in immunosuppressed individuals [1–3, 8]. Coley first described TB of the spleen in 1846, referring to an “enlarged spleen secondary to tuberculosis with absent or limited involvement of other organs” [16]. Our patient was atypical in that he was a healthy immunocompetent male without any risk factors for splenic infection, and in whom MTB diagnostic tests (WHO-approved) such as ZN staining for AFB and GeneXpert MTB/RIF were negative. The initial presentation as pyrexia of unknown origin is characteristic of splenic TB in literature [7, 17]. Apart from fever which is present in majority of patients with splenic TB, other modes of presentation reported include fatigue and weight loss, splenomegaly, [18] splenic rupture, [19] hypersplenism, portal hypertension with and without gastrointestinal bleed, and fulminant forms involving rapid progression [18]. Hematologic abnormalities include reduced cell counts but cases of polycythaemia have also been reported [20].

Isolated splenic tuberculosis frequently proves to be a diagnostic conundrum as demonstrated in our case report, mainly due to the vague non-specific nature of clinical presentation. In many case reports, imaging is important in initial identification of splenic pathology, and diagnosis further clinched by histopathological examination of a splenic fine needle aspirate, core needle biopsy or splenectomy specimen [4]. Abdominal ultrasound is a cost-effective non-invasive imaging modality in such cases and may show a miliary pattern, nodular TB, tuberculous spleen abscess, calcific TB or a combination of these findings [9]. Our patient demonstrated nodular TB. On the other hand, abdominal CT scan has added value in ruling out involvement of other organs, and has higher diagnostic accuracy than ultrasound which suffers from operator-dependence. CT scan of the abdomen would often show multiple rounded hypodense lesions which may be present in a variety of conditions other than splenic TB [1, 21]. Differential diagnosis of solitary splenic masses that ought to be considered include cysts, haematoma, fungal infection, abscesses, infarcts, vascular tumours, lymphoma and metastatic tumours [8].

Histological confirmation via splenic biopsy provides a more accurate diagnosis of splenic pathology. Aspiration cytology of splenic lesions is variable. Suri et al. [22] reported up to 88% sensitivity for fine needle aspiration cytology (FNAC) for diagnosing a tuberculous pathology in the spleen. On the other hand, microscopic observation of tissue sections allows for histological typing and staging of tubercle lesions, as well as differentiation between granulomatous lesions and other radiographically comparable lesions such as lymphomas. However literature suggests that fixing of tissues with formalin and xylene greatly reduces the sensitivity of acid-fast staining and could potentially lead to false negatives [23]. Pottakkat et al. [24] reported that AFB staining of splenic tissue sections was negative in over 339 patients who underwent splenectomy for indications other than trauma. Fukunaga et al. [23] reported that tubercle bacilli were frequently missed or underestimated with acid fast microscopy on formalin fixed, paraffin embedded tissues. In such cases, real-time polymerase chain reaction (PCR) of the tissue sections demonstrated markedly increased sensitivity over acid-fast staining.

CNB has a high diagnostic yield in splenic pathology [25] and has demonstrated superior diagnostic accuracy to FNAC in characterising splenic lesions [26, 27]. Tubercular infection is histologically confirmed by presence of typical caseation with granuloma of Langhan’s giant cells and epitheloid cells. In our case, classical granulomatous inflammation and acid fast bacilli were demonstrated upon examination of the CNB specimen and further confirmed by GeneXpert MTB/RIF.

Therefore, in apparent “TB negative” cases, performing more than one test improves yield. In our case, despite several ZN stain negative results, other diagnostic modalities were employed such as GeneXpert MTB/RIF as well as histological confirmation through tissue diagnostic procedures including FNAC and CNB. All this was done in addition to the conventional ZN staining technique to get the final diagnosis of TB spleen. It is important to note that culture is useful as well, as it is still considered the current reference standard for detecting MTB in spite of its drawbacks such as slow turnaround time (of up to 12 weeks) as well as dependence on well-equipped laboratories, technical expertise and resources which may be lacking in less developed localities [12]. In our resource-constrained setting, MTB culture is not performed regularly, although its contribution would have been invaluable in diagnostics.

Literature shows that anti-TB therapy alone may suffice in splenic TB diagnosed without splenectomy [17, 28]. Patients with splenic abscess respond well to anti-TBs [29] and a treatment duration of 6 months, similar to that for other extrapulmonary sites is recommended. A few controlled trials recommend a 12-month course of anti-TB treatment for splenic TB [19]. However, there are few reports that show inadequate or absence of response to anti-TB therapy without splenectomy [8, 30]. In such cases, splenectomy is a viable option.

The patient’s worsening symptoms on initiation of anti-TB therapy was attributed to paradoxical reaction (PR). This is defined as the “worsening of existing lesions or presentation of new lesions during anti-TB therapy” [31], or the “worsening of clinical or radiological findings following the initiation of appropriate therapy” [32]. PR is typically associated with exaggerated inflammatory symptoms including fever, [33] lymphadenitis, [34] and pulmonary manifestations [33]—as illustrated in our case by worsening fever and pulmonary disease (pleural effusion with mediastinal lymphadenopathy). It has been suggested that rapid killing of bacilli with antibiotics may lead to the release of large amounts of microbial components, which stimulate an exuberant inflammatory response, [35] and that higher baseline numbers of bacilli may potentiate this process. It has also been postulated that this is essentially a hypersensitivity reaction to persistent mycobacterial antigen [34]. Ultimately, the PR phenomenon is likely to be missed, and in such cases TB may be labelled as a misdiagnosis warranting discontinuation of therapy. Fortunately, PR is self-limiting and does not always require steroid therapy [36]. Our patient improved with supportive management and continuous anti-TBs without steroids. In summary, the chronological course of events as pertains to this case has been represented in a timeline graphic (see Additional file 1).

## Conclusions

We present a rare case of splenic TB in an immune competent host with paradoxical worsening on TB therapy. Isolated splenic TB presents a profound diagnostic complexity, especially in resource-constrained settings. Splenic core needle biopsy is indispensable in scenarios where splenectomy cannot be performed, and TB-PCR techniques are useful in ZN stain negative cases. This case highlights the need to continuously be on the lookout for TB especially in unusual presentations, including subsequent paradoxical reaction which may be encountered.

## Additional file

**Additional file 1.** Timeline of events.

## Abbreviations

AFB: acid-fast-bacilli
Anti-TB: antituberculous
CBC: complete blood count
CNB: core needle biopsy
CT: computed tomography
ELISA: enzyme-linked immunosorbent assay
FNAC: fine needle aspiration cytology
HIV: human immunodeficiency virus
PCR: polymerase chain reaction
PR: paradoxical reaction
TB: tuberculosis
WHO: World Health Organization
ZN: Ziehl–Neelsen
