# Supplementary figures and images for: Isolated splenic tuberculosis with subsequent paradoxical deterioration: a case report
Source: BMC Res Notes. 2017 Apr 24;10:162. doi: 10.1186/s13104-017-2483-2 (PMC5402664; doi:10.1186/s13104-017-2483-2)

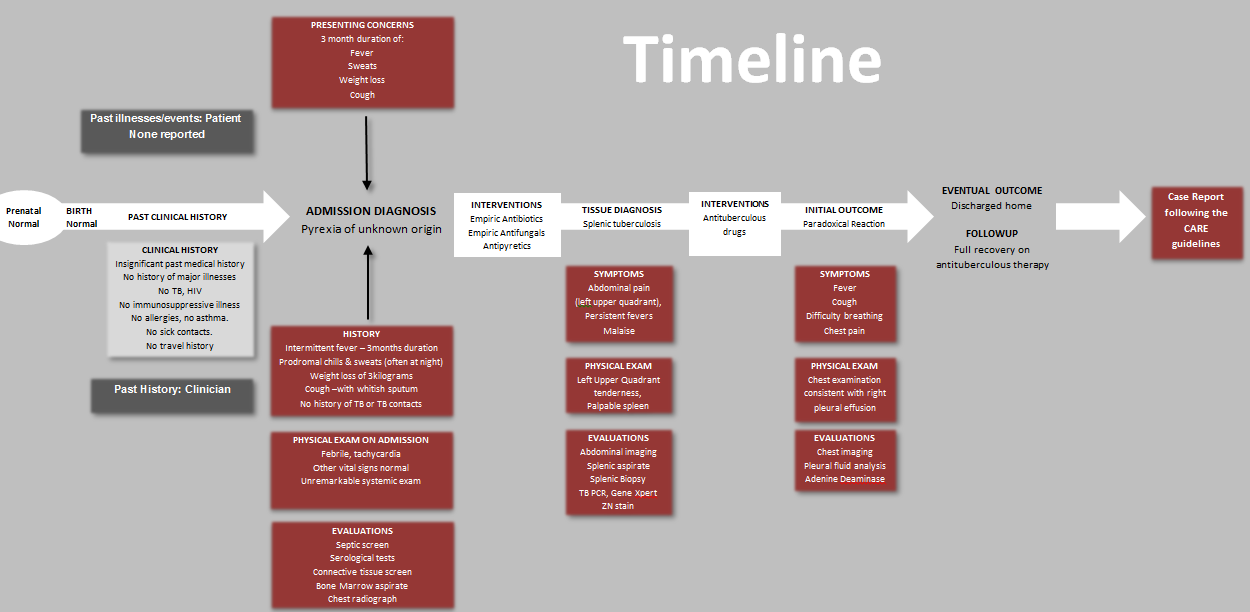

Supplement: Supplementary file 1 — Additional file 1. Timeline of events. [file 13104_2017_2483_MOESM1_ESM.docx]
